# Behavioral, Ultrastructural and Chemical Studies on the Honeydew and Waxy Secretions by Nymphs and Adults of the Asian Citrus Psyllid *Diaphorina citri* (Hemiptera: Psyllidae)

**DOI:** 10.1371/journal.pone.0064938

**Published:** 2013-06-10

**Authors:** El-Desouky Ammar, Rocco Alessandro, Robert G. Shatters Jr, David G. Hall

**Affiliations:** United States Department of Agriculture-Agricultural Research Service, Horticultural Research Laboratory, Fort Pierce, Florida, United States of America; Technion-Israel Institute of Technology, Israel

## Abstract

The Asian citrus psyllid (ACP), *Diaphorina citri* (Hemiptera: Psyllidae) is the primary vector of the bacterium causing citrus huanglongbing (citrus greening), the most serious disease of citrus worldwide. Psyllids and other hemipterans produce large amounts of honeydew, which has been used previously as an indicator of phloem sap composition and insect feeding or metabolism. Behavioral, ultrastructural and chemical studies on ACP, its honeydew and waxy secretions showed important differences between nymphs, males and females, and suggested some mechanisms by which the psyllids, especially nymphs and adult females, can minimize their contamination with honeydew excretions. The anal opening in ACP, near the posterior end of the abdomen, is on the ventral side in nymphs and on the dorsal side in adult males and females. Video recordings showed that adult males produce clear sticky droplets of honeydew gently deposited behind their body on the leaf surface, whereas adult females produce whitish honeydew pellets powerfully propelled away from the female body, probably to get their excretions away from eggs and newly hatched nymphs. ACP nymphs produce long ribbons or tubes of honeydew that frequently stay attached to the exuviae after molting, or drop when feeding on the lower side of citrus leaves. Furthermore, honeydew excretions of both nymphs and adult females are covered with a thin layer of whitish waxy material ultrastructurally composed of a convoluted network of long fine filaments or ribbons. This material is extruded from intricate arrays of wax pores in the circumanal ring (around the anus) that is found in nymphs and females but not in males of ACP or other psyllid species. Infrared microscopy and mass spectroscopy revealed that, in addition to various sugars, honeydew excretions of ACP nymphs and females are covered with a thin layer of wax similar in profile to ester waxes.

## Introduction

The Asian citrus psyllid (ACP), *Diaphorina citri* Kuwayama (Hemiptera: Psyllidae) is an invasive species that was found originally in southwestern Asia, but has now spread to many countries in South, Central and North America starting in the 1990 s [1], [2]. ACP is an economic pest of citrus, primarily because it is a vector of the phloem-limited bacteria (*Candidatus* Liberibacter spp.) associated with huanglongbing (HLB, citrus greening), currently the world’s most serious disease of citrus [3], [4]. Additionally, direct feeding damage by its piercing sucking mouthparts, as well as production of copious amounts of honeydew excretions by nymphs and adults, which leads to the growth of sooty molds, may also contribute to further economic losses in young citrus plants, especially when large numbers of ACP individuals are present [5], [6], [7].

Honeydew excretions by hemipterans are the result of feeding on the phloem sap, which has very high sugar content and osmotic pressure. Sucrose-transglucosidase activity in their gut transforms excess ingested sugar into long-chain oligosaccharides that are voided via honeydew excretion [8]. In addition to causing sooty mold growth on the host plant, which may inhibit photosynthesis [7], honeydew of psyllids and other hemipterans is known to attract many ant species [9], [10]. These ants may protect hemipteran species from their natural enemies thereby compromising biological control [11] or lead to changes to ecosystem composition and dynamics [12], [13].

Honeydew quantity or chemical analysis has been used as an indicator of insect feeding or metabolism in various hemipterans [14], [15], [16], [17], [18]. Chemical analysis of honeydew has also been used as an indicator of phloem sap composition in various host plants [19], [20], [21], in resistant versus susceptible hosts [22], [23], in diseased versus healthy plants [24], or to study within-plant variations [25], [26].

Husain and Nath [5] observed that ACP nymphs exude ‘a thick sugary liquid’ covered over with waxy secretion of the ‘circumanal glands’. These glands were described as ‘wax glands’ in *Psylla mali* nymphs and adult females by Brittain [27] using light microscopy, and in the nymphs of another psyllid, *Anomoneura mori*, by Waku [28] using light and electron microscopy. Both authors described openings of these ‘wax’ glands in the ‘anal’ or ‘circumanal ring’. Brittain [27] further indicated that these glands are found in nymphs and adult females but not in males of *Psylla mali*. However, in some previous investigations on ACP [17], [29] no distinction was made between honeydew excretions of males and females. Additionally, no chemical or ultrastructural studies have been reported on the ‘waxy’ secretions produced by nymphs and adult females of ACP or other psyllids.

The present work describes behavioral, ultrastructural and chemical studies of honeydew produced by ACP nymphs, adult males and females, as well as ultrastructure of the circumanal ring and circumanal (wax) gland openings in both nymphs and females of this economically important psyllid species.

## Materials and Methods

### Observation and Photomicrography of ACP Nymphs, Adults and their Anal Excretion Behavior

ACP nymphs and adults used here were taken from our healthy laboratory colony (not infected with Ca. L. asiaticus) that has been maintained for several generations on young healthy citrus plants (*Citrus macrophylla* Wester) in the greenhouse. Anal (honeydew) excretion behavior of ACP was observed and photographed using a stereomicroscope (Leica MZ16) fitted with a Leica DFC 320 camera, or using another stereomicroscope (Leica M60) fitted with a video camera (Leica DFC290 HD) (Leica, Switzerland). For these observations, ACP nymphs of various instars were fed in groups (10–20/group) on small pieces of fresh terminal young shoots (8–10 cm long) of sweet orange [*Citrus sinensis* (L.) Osbeck, var. Ridge Pineapple]. ACP adult males and females, separately, were also fed in groups (5–10/group) on excised young Ridge Pineapple sweet orange leaves. The cut end of each terminal shoot or leaf petiole was placed in a small (0.5 ml) microfuge tube filled with water to keep it fresh for 3–7 days. Each shoot or leaf was then placed in a 50-ml polypropylene tube (BD Falcon Conical Tubes with Flip-Top Cap; BD Biosciences, San Jose, CA) or in a Petri dish for easier observation under the stereomicroscope [30], [31]. The rearing tubes or Petri-dishes were placed on the bench top in the laboratory (at 23.7±1.5°C) with 14 hr light per day. Identification of various nymphal instars of ACP followed the drawings by Catling [32]. Honeydew excretion was observed via stereomicroscopy in hundreds of ACP nymphs of various instars and in more than 50 male and 50 female adults. Throughout this paper, ACP males and females refer to the adult stage of ACP.

Video recordings (1–2 h each) of anal (honeydew) excretion behavior of ACP males, females and nymphs as well as oviposition by females were undertaken. Video S1, provided here (1 min 52 sec. long), is composed of 4 short clips showing one male producing two consecutive excretion droplets, one on top of the other (2 separate clips), followed by one female producing one pellet (one clip), and finally another female (at lower right) producing another pellet (one clip). All clips were recorded at real time (normal speed). Since the females are much faster than males, with regard to their honeydew excretion actions, the male clips are played back at normal speed whereas the female clips are played back at a much slower speed (1/16^th^ their actual speed).

### Scanning Electron Microscopy (SEM) of ACP anal Structures, Honeydew and Waxy Secretions

**Figure 1:**
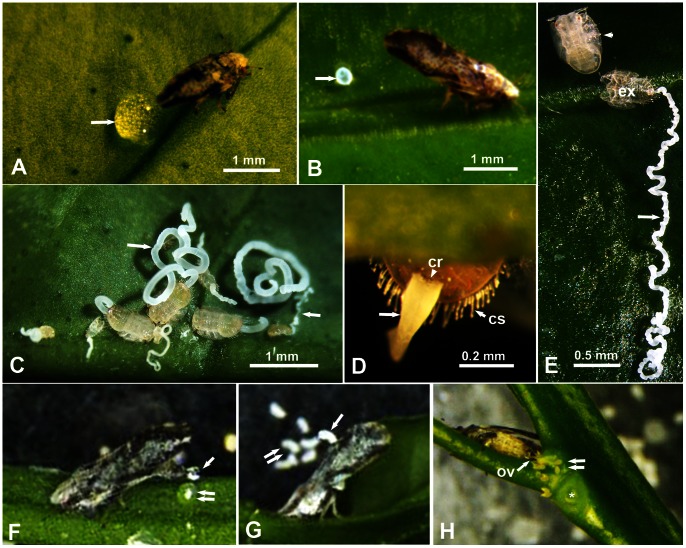
Anal excretion and oviposition behavior of the Asian citrus psyllid. **A–C**. Adults and nymphs feeding and producing their honeydew (arrows) on citrus leaves; note large clear drop produced by the male (in A), whitish pellet by the female (in B), and long whitish tubes/ribbons of nymphs (in C). **D**. Ventral view of the abdomen’s posterior end of 5^th^ instar nymph, showing honeydew (arrow) oozing out from the anus and circumanal ring (cr), and the long circumabdominal setae (cs). **E**. Newly molted nymph (arrowhead) leaving a long ribbon of honeydew (arrow) still attached to the exuvia (ex). **F–H**. Still micrographs taken from videos: **F**, a male depositing its clear honeydew droplet (arrow) gently on top of a previously excreted drop (double arrows); G, a female propelling its whitish pellet (arrow) dorsally and to the side with other previously propelled pellets (double arrows); H, a female laying eggs (double arrows), one leaf was removed at the position of the asterisk to expose eggs laid between the young shoot and the petiole of that leaf). Abbreviations: cr, circumanal ring; cs, circumabdominal setae; ex, exuvia; ov, ovipositor valvulae.

Three methods were used to prepare ACP nymphs, exuviae and/or adults for SEM to study the ultrastructure of their anal areas. The first method involved fixation and dehydration of live insects in 70% ethanol, 100% ethanol (twice) then air drying before mounting. The second method, used mainly with exuviae, was direct mounting without prior fixation or dehydration. A third method was used to clean the nymphs or adult females from their honeydew and waxy secretions, in order to examine the openings and structure of the circumanal ring (around the anus). This ‘dewaxing/cleaning’ method, modified from Lucchi and Mazzoni [33], involved soaking the insects in 100% chloroform in a small glass dish covered with a glass slide for 4 h to overnight at room temperature in a fume hood, before air drying. Honeydew excretions obtained from ACP nymphs, males and females, separately in glass Petri-dishes, were also prepared for SEM using only the second (direct mounting) method. These included the whitish, waxy honeydew tubes and pellets produced by nymphs and females respectively (Figs. 1B–D), as well as the clear honeydew droplets deposited on citrus leaves by males (Figs. 1A, F), taken with part of the leaf it was laid on. Preliminary work indicated that ACP honeydew excretions usually absorb moisture, swell, and lose their compact form if they were stored at 4°C or at room temperature under humid conditions. Thus, ACP honeydew was normally stored under vacuum for a few to several days in a desiccator, half filled with silica gel (baked at 120°C for 3 h) at room temperature, before they were processed for SEM or infrared microscopy (see below).

In all three SEM preparation methods mentioned above, the insects, exuviae or honeydew were mounted, under a stereomicroscope, on black conductive double-sided adhesive discs (9–12 mm diameter) placed on aluminum stubs (SPI Supplies, West Chester, PA), using fine-pointed forceps (Fontax no. 5; Electron Microscopy Sciences, Washington, PA). Mounted specimens were then sputter coated with Gold-Palladium for 120 sec using Hummer 6.2 Sputter Coater (Anatech USA, Union City, CA). Coated specimens were then examined at 5 or 10 Kv using a scanning-transmission electron microscope (Hitachi S-4800, Hitachi, Pleasanton, CA) in the SEM mode at magnifications of 100X to 10,000X. The number of ACP specimens examined by SEM was 8–12 waxed or dewaxed specimens in each of the following categories: males, females and nymphs. All the original electron micrographs digitally obtained in this study were automatically saved on the image management computer program (Quartz PCI version 8) associated with the Hitachi S-4800 electron microscope mentioned above.

### Infrared Microscopy and Spectroscopic Analysis of ACP Honeydew

Spectra of the honeydew produced by ACP nymphs, males and females were obtained using the Thermo Nicolet iN10 FTIR microscope in the reflection mode (for intact honeydew samples), as well as the attenuated total reflectance Fourier Transform Infrared (ATR-FTIR) mode (for crushed samples). Microscope, spectrometer, data acquisition and data processing functions were done using Thermo Nicolet Picta software. To acquire the spectra in the FTIR mode, honeydew samples were placed on an aluminum coated slide, which was mounted on the stage of the microscope. The microscope was focused on the top surface of these honeydew structures, and a linear spectra map was created along the long axis of the top surface of the tube/ribbon. Spectra were acquired at a resolution of 8 cm^−1^ averaging a total of 128 scans at each position along the map.

## Results

### Honeydew Forms and Anal Excretion Behavior of ACP Nymphs, Males and Females

ACP adult males and females, reared separately on citrus leaves or young terminal shoots, were found to feed for long periods at the same feeding site (up to 1–2 hrs), judging by their typical feeding posture, with their body angled ca. 40° with the leaf surface (Figs. 1A, B). The color and texture of anal (honeydew) excretions from males and females were markedly different. ACP males produced clear (colorless) droplets of sticky material (ca. 500–900 µm diameter) usually placed very closely behind their bodies (Figs. 1A, F). Adult females, however, produced whitish-colored, more solid and less sticky pellets of various shapes and sizes including quasi-spherical (ca. 200–300 µm diameter) and slightly curved/oblong ones (ca. 100×400 µm), usually found some distance away from their body (Fig. 1B) or at the bottom of the tube or Petri dish they were caged in (Fig. 1G). ACP nymphs of various instars, feeding on young terminal shoots, also fed for very long periods (up to several hrs) at the same feeding site on the leaf/shoot. Also while they were feeding they produced, almost continuously, a third form of honeydew excretions. These were composed of long, whitish-colored, material in the form of twisted ribbons or cylindrical, solid tubes, the length of which reached many times the body length of the nymph (Figs. 1 C–E). The width of these tubes/ribbons (ca. 30–100 µm) grew larger with each older instar (Fig. 1C). When feeding on the lower (abaxial) side of the leaf, these long honeydew excretions usually dropped on the upper surface of the leaf below or at the bottom of the caging tube or Petri dish. However, when feeding on the upper (adaxial) side of the leaf, honeydew excretions became very long, sometimes convoluted, and were usually left attached to the exuviae after molting (Figs. 1C, E).

**Figure 2:**
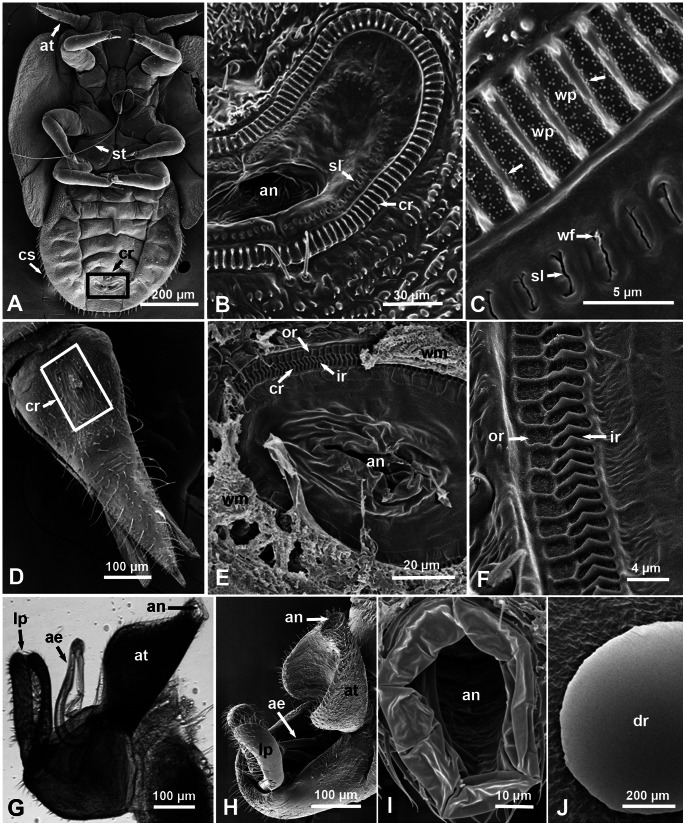
Structure of the anal area in nymphs and adults of the Asian citrus psyllid. A–F. Scanning electron micrographs (SEM) of nymphs (A–C, ventral views) and adult females (D–F. dorsal views) showing position of the circumanal ring (cr) around the anus (an) near the posterior end of the abdomen (boxed areas in A and D). In B & C (nymphs), note the ornate cuticular ridges around that ring (arrows), wax pores (wp), and the narrow cuticular slits (sl) with wax filament (wf) oozing out. In E & F (females), note the outer and inner rows (or & ir, respectively) of wax pores and the waxy material (wm) coming out of these pores (in E). **G–I.** Light and scanning electron micrographs (lateral, dorso-lateral and dorsal views, respectively) of *D. citri* males showing the anal tube (at), anus (an), aedeagus (ae), and lateral plates (lp); note lack of the circumanal ring or any cuticular ridges or wax pores around the anus. **J**. SEM of a male’s honeydew droplet (on a citrus leaf) showing no filamentous structures on the surface like those found on the honeydew of nymphs or females. Other abbreviations: at, antenna; cs, circumabdominal setae; st, stylets.

The process of producing anal/honeydew excretions by ACP males and females was recorded by video (Video S1). Although the anal opening of both males and females is located on the dorsal side near the end of the abdomen (Figs. 2D, G–I), the behavior of anal excretion in males and females differed considerably. Males began by bending the end of their abdomen downward, thus changing the position of the anus from upward to downward, before gently laying a clear droplet of honeydew immediately behind their bodies. This process took about 2–3 seconds, which was repeated every 4 minutes or longer. Frequently an individual male was observed to layer several honeydew droplets on top of each other (Fig. 1F and Video S1), apparently until this honeydew pile became too large, then the male moved away to feed and excrete at another site on the leaf. The female, on the other hand, produced each honeydew pellet at a much faster speed (less than 1/4^th^ of a second) but the time that elapsed between producing pellets was much longer (20–30 min or longer). Video S1 (with playback slowed down 16 times) shows that the ACP female starts by twitching its wings briefly, bending the end of the abdomen slightly downward, then pushes its honeydew pellet dorsally through the pyramid-shaped folded wings. She leaves this pellet momentarily held between the wings, while she bends the abdomen downward again, then uses the end of the abdomen (with a strong thrust at a very high speed) to propel the pellet upward and sideways. The pellet normally did not fall behind the female body, but usually it fell some distance away on the right side of the female body (Fig. 1G and Video S1). Other video recordings (not included here) showed that ACP females lay their eggs, using their ovipositor at the end of the abdomen, immediately behind their bodies, on young feather leaves or in the axillary corners between young terminal shoots and leaf petioles (Fig. 1H).

### Ultrastructure of the Circumanal Ring and Wax Gland Openings in ACP Nymphs and Adults

**Figure 3:**
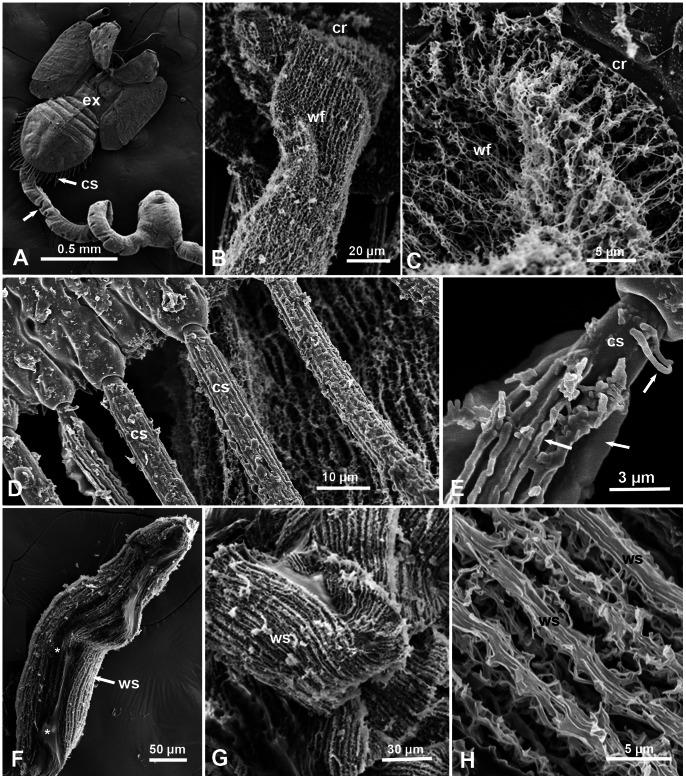
Scanning electron micrographs of waxy structures on the surface of honeydew and circumabdominal setae of the Asian citrus psyllid. **A.** Honeydew tube (arrow) attached to the exuvia (ex) of a fifth instar nymph (dorsal view); note long circumabdominal setae (cs) around the abdomen. **B & C**. Higher magnifications of the wax filaments (wf) found on the surface of the nymphal honeydew oozing out of wax pores in the circumanal ring (cr). **D & E**. Details of the bases of the circumabdominal setae (cs) of 5^th^ instar nymphs, showing the waxy material covering these setae (arrows). **F**. Adult female pellet (slightly open at the center) showing the wax structures (ws) on the surface, and gluey material devoid of filamentous structures inside (asterisks). **G & H**. Higher magnifications of the wax structures (ws) as filaments or ribbons on the surface of female pellets.

In ACP nymphs, the circumanal ring (around the anus) is located on the ventral side near the end of the abdomen (Fig. 2A). It is somewhat crescent-shaped, with an anterior concave side and a posterior convex one (Figs. 2A, B). In 3^rd^– 4^th^ instar nymphs this ring measured about 110–130 µm long, and 30–40 µm wide. At the ultrastructural level, SEM showed that the cirucmanal ring is composed of prominent cuticular ridges (5–7 µm long, and 0.4–0.7 µm wide). The wax pores between each ridge and the next (1.6–1.7 µm wide) are full of small dot-like structures (probable mini-pores) arranged in sets of 3 producing a triangular arrangement (Fig. 2C). Inside this ring of ridges and wax pores, another ring of narrow open cuticular slits (each ca. 2.4–2.6 um long and up to 0.2 um wide) was found (Figs. 2B, C). In some cases, thin filaments of secretions could be seen oozing out from these slits (Fig. 2C). The wax pores between the ridges as well as these narrow slits apparently are the openings through which the circumanal (wax) glands under the cuticle (described in *P. mali* by Brittain [27]) produce their waxy secretions (Figs. 2C, 3B, 3C).

Around the edge of the abdomen in ACP nymphs, is a row of long setae, normally covered with waxy material, the length of which increased in older instars (Figs. 1D, 2A, 3A, 3D–E). Their numbers also increased with each instar as follows: 1^st^ instar, 10–12 setae; 2^nd^ instar, 15–17 setae; 3^rd^ instar, 30–38 setae; 4^th^ and 5^th^ instars, 46–56 setae (with some overlap between the last two instars). One function of these setae appears to be keeping the excreted honeydew tubes/ribbons in a somewhat straight line so that they do not stick to the abdomen’s end (Figs. 1C, D). However, in some cases, these long tubes may bend above or somewhat away from the body (Fig. 1C). These ‘circumabdominal’ setae were not found in ACP adult males or females.

In ACP females, the cirumanal ring (also around the anus) is found on the dorsal side at the anterior third of the dorsal plate of the genital segment (Fig. 2D). It is oblong in shape (ca. 100×65 µm in diameter) with its wider diameter parallel to the insect body (Figs. 2D, E). At the ultrastructural (SEM) level, this ring is composed of two rows of wax pores with ornate cuticular ridges, the outer row has shallow ridges around almost square pores (each 1.5–2.2 µm wide), and the inner row has deeper vertical ridges around elongated pores (Figs. 2E, F). Each of the inner ridges has 90° corners, about 2 µm long and 2 µm deep, with ca. 1 µm wide wax pores in between. No open slits in the cuticle, like those found inside the nymphal circumanal ring, were found in the female ring. However, smaller dot-like structures similar to those found in the nymphal wax pores, but not arranged in triangles, were found in both the inner and outer rows of wax pores in the female (Fig. 2F).

The anal opening in ACP male is also located on the dorsal side (as the female) on top of the anal tube (Figs. 2G–I). But it is structurally much simpler and does not have any circumanal ring with cuticular ridges, wax pores or slits like those found in ACP females or nymphs (Figs. 2A–F).

### SEM Ultrastructure of the Honeydew in ACP Nymphs and Adults

At the ultrastructural level, using SEM with magnifications of 500–10,000x, the outer surface of the honeydew tubes or ribbons of ACP nymphs, was composed of very long, extremely fine, convoluted filaments that apparently came out of the wax pores and cuticular slits described above in the circumanal ring of nymphs (Figs. 3A–C). Waxy structures were also found by SEM covering the circumabdominal setae of the nymphs (Figs. 3D, E.). Honeydew pellets of adult females also were covered, on the outside, with long thin filaments or ribbons that were normally wider than those of the nymphs, and also appeared to be coming out of the wax pores described above in the circumanal ring of females (Figs. 2E, 3F–H). On the other hand, SEM of honeydew droplets of adult males had a smooth surface (Fig. 2J), with no waxy/filamentous structures similar to those found on the surface of honeydew of nymphs and females.

### Infrared and Spectroscopy Analysis of Honeydew of ACP Nymphs and Adults

**Figure 4:**
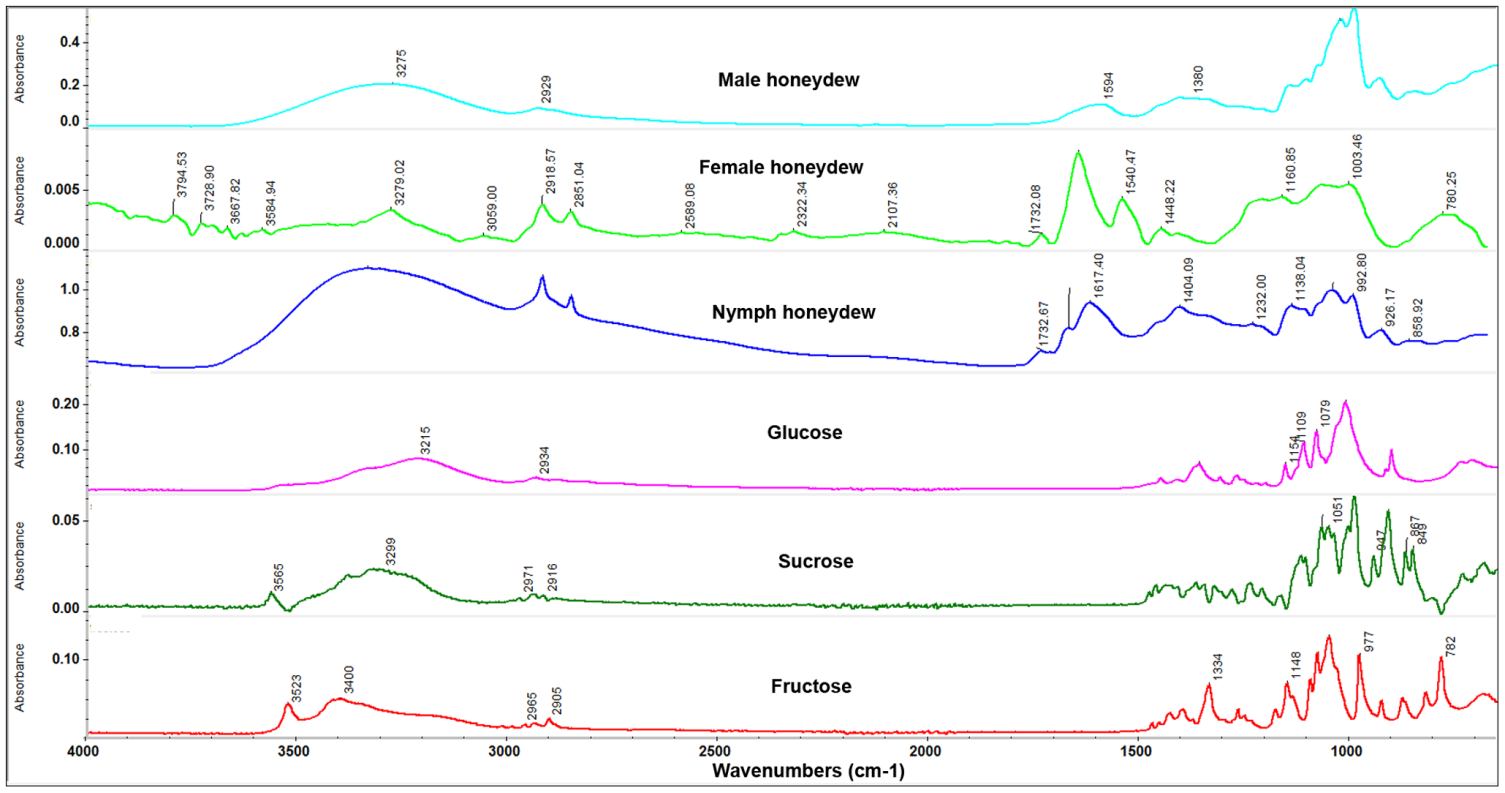
FTIR microscope reflection spectra of the surface of male, female and nymphal honeydew excretions (top three panels) compared to attenuated total reflectance FTIR spectra of typical monosaccharides. The honeydew spectra contain many peaks attributable to simple saccharides, especially the broad peak centered at 3300 cm^−1^ attributable to the O–H bonds in sugars and water, and broad poorly resolved peaks at frequencies lower than 1500 cm^−1^ which indicate a mixture of the saccharides whose spectra appear in the lower three panes. However, the peaks at 2850 and 2910 cm^−1^ are more pronounced in the honeydew and the peak at 1733 cm^−1^ does not appear in the saccharide spectra. The male excreta show spectra typical of aqueous sugars.

Preliminary attempts using attenuated total reflectance Fourier Transform Infrared (ATR-FTIR) spectra of ACP honeydew (in which the samples were crushed on the diamond ATR crystal and then scanned) showed no sign of wax being present in the honeydew of nymphs, males or females. Typically, ATR-FTIR analysis of these excretions indicated that this material is composed mainly of water and sugars. The spectra are characterized by huge broad bands in the region from 3600–2800 cm^−1^, attributed to water and hydroxyl groups, and other large bands from 1100 to 1000 cm^−1^ attributed to the carbon-oxygen single bonds of sugars, including sucrose, fructose and Beta-D-(+)-glucose (Fig. 4). Peaks in the region from 2850 to 3050 cm^−1^ due to C–H bonds were weak and ill-defined, which is characteristic of wet sugar samples.

**Figure 5:**
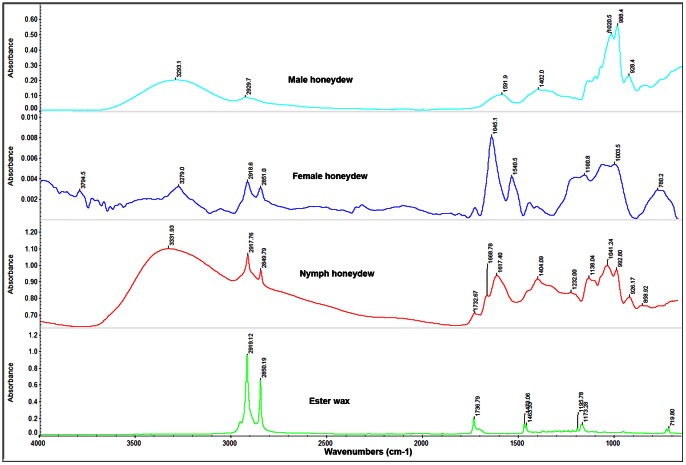
FTIR microscope reflection spectra of the surface of male, female and nymphal honeydew (top three panels) compared to attenuated total reflectance FTIR spectrum of beeswax, a typical ester wax. While all the honeydew spectra are dominated by saccharide peaks (as shown in Fig. 4), the female and nymphal honeydew spectra are markedly different from that of the male. Peaks at 2850 and 2910 cm^−1^ (which are attributable to C–H bonds in a long chain aliphatic molecule) and the peak at 1732 cm^−1^ (which is attributable to the C = O of an ester carboxyl group) strongly indicate the presence of an ester wax on the surface of honeydew of females and nymphs.

Given these findings, we reasoned that if wax was present in the honeydew of ACP females and nymphs, as our SEM studies above suggested, it must be only on the surface of the honeydew pellets or tubes produced by females and nymphs respectively. Thus, the samples were subjected to FTIR reflectance microscopy, in which intact honeydew samples were not crushed but simply scanned after the microscope is carefully focused on the upper surface of the honeydew structures. While the large peaks mentioned above (typical of sugars) also existed in the FTIR reflectance microscopy spectra of the top/surface of the honeydew of males, females and nymphs (Fig. 4), spectra of the female and nymphal honeydew also displayed peaks in the 1735–1745 cm^−1^ range attributed to the carboxyl C = O of the wax esters as well as two pronounced peaks at 2850 and 2920 cm^−1^ attributed to C–H bonds of aliphatic hydrocarbons, fatty and ester waxes such as bees wax (Fig. 5). No pronounced peaks typical of bees or ester waxes were found in FTIR spectra of the surface of ACP male honeydew (Fig. 5).

## Discussion

Feeding on the phloem presents certain challenges to hemipteran insects, which normally have certain adaptations to counter these challenges. First, the high sugar content and osmotic pressure of phloem sap is countered by sucrose-transglucosidase activity in their guts, which transforms excess sugar into long-chain oligosaccharides voided as honeydew excretion [8]. This, however, presents another problem for these insects: how to avoid being contaminated or even drowned by their own sticky, sugar-rich, honeydew [34], especially for the more vulnerable eggs and young nymphs.

ACP produces copious amounts of honeydew excretions by nymphs and adults [5], [6]. Although it has been suggested earlier that these excretions are covered with ‘waxy material’ [5] the chemical or ultrastructural composition of this material, as well as the fine structure of the wax gland openings in ACP or other psyllids have not been reported earlier. Previous investigations on ACP adult honeydew indicated that its major components were: sucrose, D-fructose, mannose, trehalose, myo-inositol, ribitol, galactose, quinic acid, and malic acid [29]. In our present work, we show that, in addition to various sugars, the honeydew of ACP nymphs and adult females are covered by a thin layer similar in its IR spectrum to those of bees wax and other ester waxes. The chemical composition of waxes produced by Hemiptera have been investigated mainly in whiteflies and scale insects. The surface lipids on nymphs and exuvia of several whitefly species (Aleyrodidae) contained largely wax esters, long-chain aldehydes, hydrocarbons and long-chain alcohols [35], [36], [37], [38].

The wax (circumanal) glands in nymphs and female adults of the apple psyllid (*P. mali*) were described at the light microscopy level by Brittain [27] as ‘lobular masses directly beneath the cuticle, consisting of tall columnar epithelial cells with a well defined nucleus at the base and frequently a space filled with secretion between the cells’. Waku [28], studied wax glands in nymphs of another psyllid (*A. mori*) by transmission electron microscopy, and indicated that these glands consisted of two kinds of cells, derived from epidermal cells: wax cells, which produce and secrete the wax, and flat interstitial cells found among these cells. Each wax cell has a long, wide duct which opens at the cuticle. The openings of the wax glands in the circumanal ring of psyllid nymphs and females were also described at the light microscopy level by Brittain [27] and Husain and Nath [5] in *P. mali* and *D. citri*, respectively. It is surprising how accurate these descriptions were, given the resolution of the light microscopy when compared with SEM. However, our SEM work here provided much finer details of the wax pores and cuticular slits found on the circumanal ring of ACP females and/or nymphs. The wax pores we described here, especially in ACP nymphs, are somewhat ultrastructurally similar to openings of the wax glands, described as ‘canaliculated cuticle’, in the nymphal anal ring of the psyllid *A. mori* [28]. But they are considerably different from the ‘wax pores’ found on wings of the flatid planthopper *Metcalfa pruinosa* (Fulgoroidae) [33], or from ‘pores in the wax gland plates’ on the cuticle of some aphid species [39].

The primary role of the wax layer on the honeydew of ACP nymphs and females is likely to limit/minimize the contamination of nymphs and eggs by the thick, sticky and sugary honeydew. Similarly, Smith [39] suggested that the primary role of the secreted wax on the surface of aphid cuticle is to prevent the aphids becoming contaminated by their own honeydew as well as that of other members of a colony. This may also be the purpose of the difference in behavior, reported here, between ACP males and females in their honeydew excretion behavior. Males deposit their honeydew droplets, devoid of waxy material, immediately behind them, whereas the females cannot do the same (otherwise they will likely smother their deposited eggs and newly hatched nymphs with this sticky material). Hence, females not only produce wax-covered excretions but also propel them some distance to the side away from their bodies, their eggs, and newly hatched nymphs. Heavy feeding and honeydew production by nymphs or adults of another psyllid (*Ctenarytaina thysanura*) greatly reduced the attractiveness of terminal shoots for oviposition [40]. This deterrence was associated with the production of honeydew resulting in the development of sooty moulds**.** That ACP nymphs and females have certain mechanisms and adaptations to avoid contamination with honeydew whereas ACP males apparently do not is interesting, and suggests that ACP males and females may occupy different sites on citrus plants, especially with regard to egg laying. ACP females normally lay their eggs on very young, feathery and folded leaves, or in the axillary corners between young terminal shoots and leaf petioles (Fig. 1H) [2], [5], [6], [7], whereas males were not normally observed feeding on these sites (data not shown).

In addition to the wax covering of their honeydew excretions, ACP nymphs seem to have developed two more ways to minimize contamination with honeydew. First, the wax-covered circumabdominal setae (around the abdomen), found in nymphs but not in adults of either sex, appear to keep the long honeydew ribbons or tubes away from their bodies (Figs. 1C, D & 3A, D). These setae were described earlier by light microscopy in ACP nymphs as ‘lanceolate setae’ by Husain and Nath [5], who also indicated that they were covered with narrow tubular sheaths of waxy secretion, produced by glands situated around their bases. Second, we observed that most of the nymphs feeding on the upper side of the leaf usually leave their long honeydew tubes or ribbons behind attached to the exuvia during molting (Fig. 1E). In the field, honeydew tubes or ribbons are infrequently noticeable (unpublished observations), perhaps due to wind or wind-induced movement of citrus leaves. In addition to reducing contamination with honeydew, it has been suggested that waxy secretions in aphids may also provide ‘a microclimate coat’ or afford some protection against fungus, parasite or predator attacks [39]. But this may be true mainly with insects that produce wax filaments on larger areas of their cuticle, not just their circumanal ring or circumabdominal setae, like mealybugs, scale insects, some aphids and planthoppers [33], [34], [39], [41], [42].

The phloem feeding scale insects and mealybugs are known to posses several mechanisms to limit contamination from their own sticky honeydew excretions [34], [41], [42]: 1. filaments of wax often coat the insect body so that honeydew droplets do not adhere to it; 2. the anal opening is surrounded by an anal ring that usually bears setae and pores, wax from these pores coats the anal ring setae and prevents honeydew droplets from sticking to them; and 3. the anal ring has certain ways by which it can propel the honeydew away from the inset body. Our study shows that ACP nymphs may have mechanisms similar to the first two mentioned above, but the wax produced is on the surface of honeydew excretions and circumabdominal setae rather than on the insect body itself. We also show that ACP females posses something similar to the third mechanism, i.e. propelling their wax-covered excretions to the side away from their bodies. To our knowledge, this behavioral difference between males and females has not been described earlier for ACP or other psyllid species. We hope that this investigation furthers our understanding of the biology, adaptations and survival of this group of hemipteran insects that is economically important on many field and horticultural crops worldwide.

## Supporting Information

Video S1 **Anal (honeydew) excretion behavior of Asian citrus psyllid males and females.** This video (1 min 52 sec. long) is composed of 4 short clips showing one male producing two consecutive, clear, honeydew drops, one on top of the other (2 separate clips), followed by one female producing one, yellowish/whitish, honeydew pellet (one clip), and finally another female (at lower right) producing another pellet (one clip). All clips were recorded at real time (normal speed); the male clips are played back at normal speed, whereas the female clips are played back at a much slower speed (1/16^th^ their actual speed).
(WMV)
